# Neoadjuvant targeted immunotherapy followed by surgical resection versus upfront surgery for hepatocellular carcinoma with macrovascular invasion: A multicenter study

**DOI:** 10.7150/jca.94539

**Published:** 2024-04-08

**Authors:** Xiang'an Wu, Yuxin Wang, Sen Wang, Ye Chen, Jiashu Han, Chao Wang, Meng Zhang, Xiongwei Hu, Biao Song, Xueshuai Wan, Haifeng Xu, Haitao Zhao, Xin Lu, Yilei Mao, Xinting Sang, Zhixian Hong, Xiaoyong Wei, Shunda Du

**Affiliations:** 1 Department of Liver Surgery, Peking Union Medical College Hospital, PUMC and Chinese Academy of Medical Sciences, Dongcheng, Beijing 100730, China.; 2 4+4 Medical Doctor Program, PUMC and Chinese Academy of Medical Sciences, Dongcheng, Beijing 100730, China.; 3 Department of Hepatobiliary Surgery, the 302nd Hospital of Chinese PLA, Fengtai, Beijing, 100039, China.; 4 Department of Hepatobiliary Surgery, Jiangxi Provincial Cancer Hospital, Nanchang, Jiangxi, 330029, China.; 5 Department of Hepatobiliary Surgery, the Fourth Affiliated Hospital of Hebei Medical University, Shijiazhuang, Hebei, 050011, China.

**Keywords:** targeted immunotherapy, surgery, complications, the recurrence rate

## Abstract

**Background:** This study aimed to investigate the safety and efficacy of preoperative targeted immunotherapy followed by surgical resection for hepatocellular carcinoma (HCC) patients with macrovascular invasion.

**Method:** Clinical information of HCC patients with macrovascular invasion was collected from four medical centers. These patients were divided into two cohorts: the upfront surgery group (n=40) and the neoadjuvant group (n=22). Comparisons between the two groups were made with appropriate statistical methods.

**Results:** HCC Patients with macrovascular invasion in the neoadjuvant group were associated with increased incidence of postoperative ascites (72.73% vs. 37.5%, P=0.008), but shorter postoperative hospital stay (10 days vs. 14 days, P=0.032). Furthermore, targeted immunotherapy followed by surgical resection significantly reduced the postoperative recurrence rate at both 3 months and 1 year (9% versus 28.9%, 32.1% versus 67.9%, respectively; P=0.018), but increased the postoperative nononcologic mortality rate within 1 year (20.1% vs. 2.8%; P= 0.036).

**Conclusion:** For HCC patients with macrovascular invasion, preoperative targeted immunotherapy significantly decreased the postoperative tumor recurrence rate while maintaining relative safety, but such a treatment may also result in chronic liver damage and increased risk of nononcologic mortality.

## Background

Among all malignant tumors, primary liver cancer ranks 6th in incidence and 3rd in mortality 1. For early-stage hepatocellular carcinoma (HCC), radical surgery is the primary treatment with a 5-year survival rate of 60-70% 2. However, more than 50% of HCC patients are in the Barcelona Clinic Liver Cancer (BCLC) stage C and D at the time of diagnosis. Late diagnosis deprives most patients of surgical opportunities and leads to a high mortality rate of HCC 3. According to the diagnostic criteria of the 2019 edition of the Chinese clinical guidelines for primary liver cancer, invasion of the intrahepatic vessels, including the portal vein and hepatic vein, defines China Liver Cancer (CNLC) stage Ⅲa and progressive HCC, equivalent to the partial BCLC stage C HCC 4, 5. Surgical resection for such patients leads to a better prognosis, with overall survival (OS) of 8.9-33 months compared to 4-6 months of untreated patients 6. Therefore, radical surgical resection is currently recommended for HCC patients with macrovascular invasion, but high postoperative recurrence still impairs the prognosis of some patients 7-9. Preoperative adjuvant therapy for HCC patients with macrovascular invasion may increase the rate of radical surgical resection, reduce the risk of postoperative recurrence, and prolong OS 10-12. For instance, Xubiao Wei et al. found that neoadjuvant radiotherapy (RT) provided significantly better postoperative survival outcomes than surgery alone for resectable HCC with portal vein tumor thrombosis (PVTT) 13. Jianping Zhao et al. suggested the neoadjuvant drug-eluting bead transarterial chemoembolization (D-TACE) and tislelizumab therapy were safe and benefited to the pathological results and prognosis for patients with resectable or borderline resectable HCC 14.

In recent years, targeted immunotherapy has shown great advantages in treating HCC, leading to an improved conversion rate of unresectable HCC, diminishing postoperative recurrence and prolonging OS 15-17. For example, a phase Ib clinical trial showed that the combination of lenvatinib and pembrolizumab for unresectable HCC achieved a median OS of 22 months and an objective response rate (ORR) of 46%, despite grade≥3 treatment-related adverse events (AEs) occurring in approximately 67% of patients, which were mostly manageable 18. The IMbrave150 global multicenter phase Ⅲ trial showed that the combination of atezolizumab and bevacizumab, compared to single-agent sorafenib, significantly benefited the survival of patients with unresectable HCC, with a 12-month survival rate of 67.2% versus 54.6% and an improved median progression-free survival (PFS) of 6.8 months versus 4.3 months. Common adverse effects included hypertension, proteinuria, liver dysfunction, diarrhea, and decreased appetite 19. A study of 38 patients with advanced HCC with extrahepatic oligometastasis by Yang et al. reported that the combination of tyrosine kinase inhibitors (TKIs), immune checkpoint inhibitors (ICIs) and at least one local therapeutic modality enableed successful tumor downstaging and subsequent surgical resection in 9 patients (23.7%), with a low recurrence rate during the follow-up period. All patients had varying degrees of AEs, with approximately 55.6% being grade 3 AEs 20. Other ICIs, such as durvalumab, and nivolumab, have also been shown to significantly prolong OS in advanced HCC, with manageable adverse reactions 21, 22.

Targeted immunotherapy has achieved great success in the treatment of advanced HCC. Previous studies reported prolonged OS, tumor downgrading, lower risk of postoperative recurrence, and tolerable AEs associated with targeted immunotherapy 23-25. HCC with macrovascular invasion is an advanced-stage malignancy that may benefit from targeted immunotherapy and subsequent surgical resection 26, but the comparison between surgery following neoadjuvant targeted immunotherapy and upfront surgery in terms of short-term postoperative complications and long-term prognosis remains unclear. Therefore, this multicentered study aimed to investigate the safety and efficacy of targeted immunotherapy followed by surgical resection for HCC with macrovascular invasion, taking upfront surgery as control.

## Patients and Methods

### Patients

This study conducted a retrospective analysis of clinical data collected from HCC patients with macrovascular invasion who underwent hepatic surgery between January 2019 and May 2022 at four medical institutions: Peking Union Medical College Hospital, Beijing 302 Hospital, Jiangxi Provincial Cancer Hospital, and the Fourth Affiliated Hospital of Hebei Medical University. Enrollment criteria included: a) adults aged 18 and 70 years with a clinical or pathological diagnosis of HCC; b) evidence of tumor thrombus in the portal or hepatic vein ascertained through imaging or postoperative pathology, corresponding to CNLC stage IIIa or a subset of BCLC stage C HCC. The HCC diagnostic and staging guidelines were in accordance with Chinese standards 4, 5. c) technically and oncologically resectable HCC; d) HCC patients with macrovascular invasion who meet the general condition and liver function requirements for surgery according to the preoperative assessment and have undergone complete tumor resection. Exclusion criteria were as follows: a) HCC previously treated with other antitumor therapies excluding combined targeted immunotherapy; b) HCC with distant extrahepatic metastases; c) recurrent HCC; d) a history of other malignancies.

Informed consent was obtained from all enrolled patients, and approval was obtained from the ethics committees of the four medical centers mentioned above. Demographic, surgical, pathological and postoperative information were collected from enrolled patients. The dimensions and multiplicity of tumors were determined by the last preoperative imaging. A major liver resection was defined as surgical removal of three or more liver segments. The treatment interval refers to the duration between the cessation of targeted or immunotherapy agents and the date of surgery; typically, ICIs are discontinued 4 weeks prior to surgical intervention.

### Treatments

Patients were divided into two treatment cohorts: the upfront surgery group and the neoadjuvant group. The former underwent immediate surgery, while the latter received targeted immunotherapy, potentially supplemented with additional adjuvant treatments, such as TACE, Hepatic Artery Infusion Chemotherapy (HAIC), and radiothrapy (RT), prior to surgery. All neoadjuvant treatments were based on the combination of antiangiogenic agents and ICIs, but the specific regimens were personalized for each patient. Tumors were monitored monthly by enhanced Computed Tomography (CT) or Magnetic Resonance Imaging (MRI) during the preoperative period. The Response Evaluation Criteria in Solid Tumors (RECIST) version 1.1 and modified RECIST (mRECIST) criteria were used to assess treatment responses. Shrinkage or stabilization of the tumors suggested surgery, which would be individualized according to each patient's condition 27.

During the surgery, Pringle's maneuver was employed to obstruct the hepatic blood inflow, thereby minimizing intraoperative bleeding. Postoperative complications were stratified according to the Clavien-Dindo (CD) classification 28, with principal complications including postoperative hemorrhage, liver dysfunction, bile leakages, pulmonary infections, alterations in plasma albumin levels, bilirubin concentrations, and prothrombin time (PT). Complications were considered severe if classified as grade 3 or above according to the CD classification.

Enrolled patients were followed-up until June 2022. The primary endpoint of was recurrence-free survival (RFS), which was defined as the interval from the date of surgery to the detection of tumor recurrence by radiological examination. The secondary endpoint was OS, defined as the period from surgery to death, loss of follow-up or the end of the follow-up period. Postoperative complications were recorded during the postoperative hospital stay. Patients underwent regularly followed-ups, with monthly check-ups in the first six months after the surgery, quarterly for the subsequent two years, and biannually thereafter. Monitoring for postoperative recurrence involved alpha-fetoprotein (AFP) blood tests, hepatobiliary ultrasounds, and CT or MRI scans. Tumor recurrence was confirmed by a rise in AFP levels or visible lesions on imaging.

### Statistics

Continuous variables were analyzed using median values and ranges with the Mann-Whitney U test, while categorical variables were expressed as percentages and analyzed with the chi-square test. Competing risk models were employed to explore the effect of upfront surgery versus neoadjuvant therapy on postoperative RFS. P <0.05 was considered statistically significant. All statistical analyses were performed using IBM SPSS Statistics 22.0, GraphPad Prism 8, and R version 4.13.

## Results

### Characteristics

From January 2019 to May 2022, a cohort of 62 HCC patients with macrovascular invasion who underwent liver resection in the four participating medical centers was identified as eligible for the study. Of these, 40 underwent immediate surgery, and 22 received surgical resection following targeted immunotherapy. The average duration of preoperative adjuvant therapy was 4 months (95% confidence interval [CI] 3.0-4.9), with a median of 18 days elapsing between treatment conclusion and surgery (95% CI: 9.5-27.0) (Table 4). The baseline clinical characteristics of the patients in both groups are shown in Table 1. More than 90% of all patients were males of similar age, with 83.9% having a history of hepatitis B virus (HBV) infection. Fifteen percent of the upfront surgery group and 50% of those receiving neoadjuvant therapy were on regular preoperative antiviral treatment. All patients had an Eastern Cooperative Oncology Group (ECOG) performance status of 0-1, and 93.5% presented with a preoperative liver function of Child-Pugh A. Median baseline levels of total bilirubin, albumin, and PT were within the normal limits for both groups, and elevated AFP was observed in over 60% of patients. Radiological examinations revealed that both groups had predominantly solitary tumors of similar size. The primary type of tumor thrombus was PVTT, and the main surgical procedure was anatomical hepatictomy. There was a higher proportion of minor hepatic resections within the upfront surgery group, whereas major hepatic resections were more common in the neoadjuvant group.

**Figure 1:**
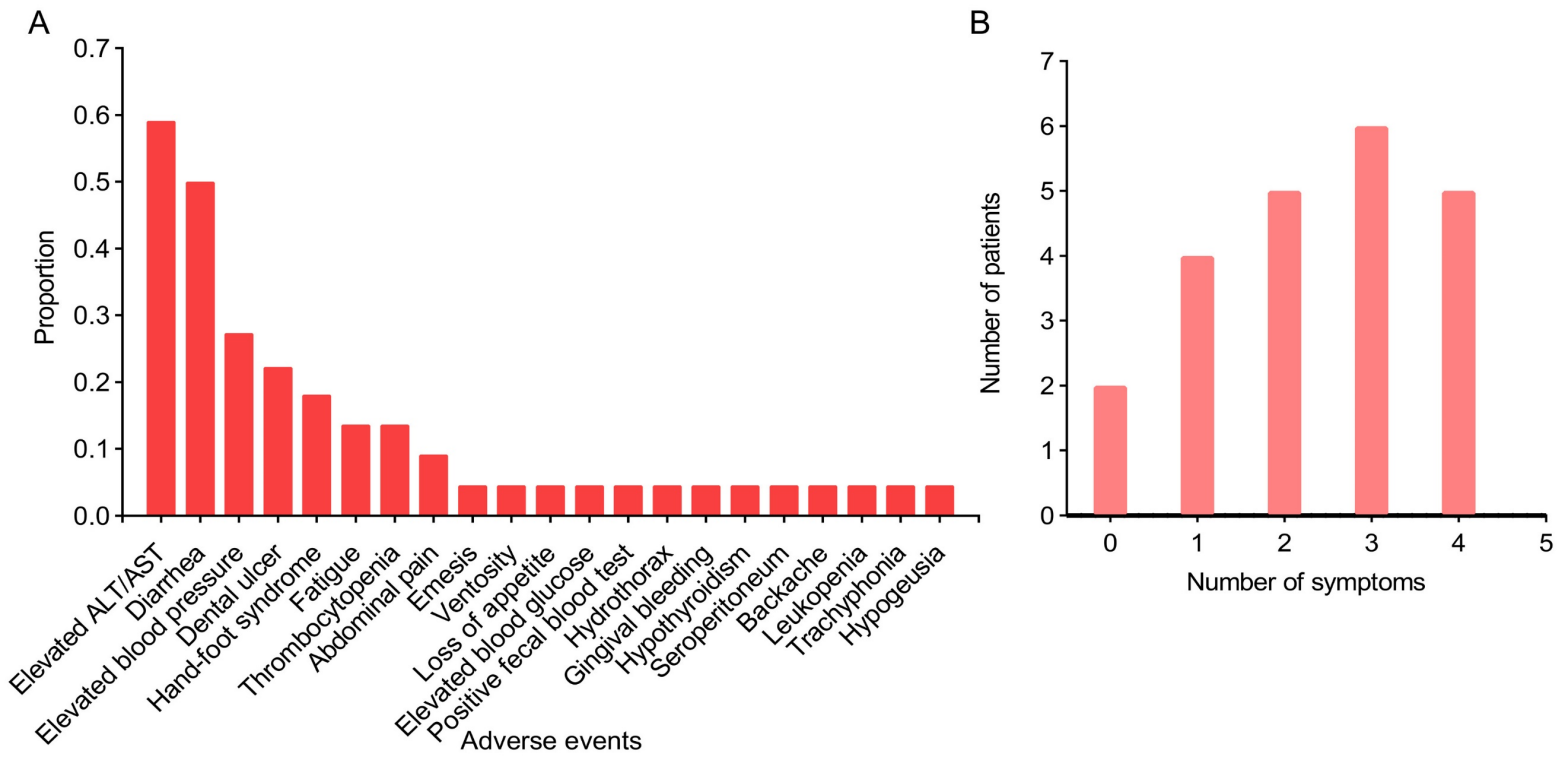
Overview of adverse events (A) and distribution of symptoms (B) in patients with preoperative targeted immunotherapy. A, the x-axis represents the various adverse events, and the y-axis represents the proportion of adverse events to the total cases; B, the x-axis represents the number of symptoms one has experienced, and the y-axis represents the number of patients.

In this study, six preoperative adjuvant targeted immunotherapy regimens were utilized (Table 4): Antiangiogenic+ICI for two patients, Antiangiogenic+ICI+HAIC for four patients, Antiangiogenic+ICI+TACE for eight patients, Antiangiogenic+ICI+TACE+HAIC for one patient, Antiangiogenic+ICI+RT for three patients, and Antiangiogenic+ICI+TACE+RT for four patients. The combination of Antiangiogenic + ICI with TACE or HAIC represented the most employed therapeutic protocol, used in 13 out of 22 cases. Upon preoperative evaluation, 21 out of 22 patients attained preoperative partial remission (PR), and 1 out of 22 exhibited stable disease (SD). The postoperative pathology indicated that pathologic complete response (PCR) occurred in 3 patients. Comparison between the six regimens revealed that the antiangiogenic+ICI+HAIC arm had the highest proportion of PCR (2/4). The most common AE of targeted immunotherapy was aminotransferase (ALT) elevation (59.1%), followed closely by diarrhea (50%) (Figure 1A). Other common AEs included elevated blood pressure, dental ulcer, hand-foot syndrome, fatigue, thrombocytopenia, abdominal pain, emesis, and ventosity, etc. The variety and frequency of symptoms exhibited considerable interpatient variability: some individuals presented with no symptoms or a singular symptom, whereas others experienced multiple complications concurrently (Figure 1B). All AEs were tolerable and manageable with supportive therapy and symptomatic relief, resulting in no treatment discontinuations or transitions to alternative antitumor therapies.

Information regarding the surgical procedures and short-term postoperative outcomes is summarized in Table 2. The median operative time and hilar occlusion duration for the upfront surgery cohort were 240.0 minutes (interquartile rage [IQR] 202.5-300.0) and 21.5 minutes (IQR 12.3-30.8). respectively. In contrast, the neoadjuvant group recorded longer median times of 292 minutes (IQR 232.5-343.8) for surgery and 31.5 minutes (IQR 0.0-62.3) for hilar occlusion; however, these differences did not attain statistical significance. Interestingly, the neoadjuvant group exhibited lower median intraoperative blood loss [300.0 ml (IQR 175.0-575.0 ml) versus 400 ml (IQR 200.0-600.0 ml)], yet a greater proportion of these patients required perioperative blood transfusions (63.6% versus 45%). This heightened demand for blood products in the neoadjuvant group may be attributable to preoperative adjuvant therapy-related hepatic function compromise. Neoadjuvant group patients also experienced a significantly higher incidence of postoperative massive ascites (72.73% versus 37.5%; P=0.008). There was no significant difference in postoperative serum albumin, total bilirubin, and PT levels across both groups, possibly because of the benefits of supportive measurements, such as exogenous albumin supplementation and plasma transfusions, etc.

More reassuringly, the neoadjuvant group demonstrated CD classifications (P=0.669), rates of major postoperative complications (7.5% vs. 18.2%; P=0.204), and incidences of postoperative biliary leakages (7.5% vs. 13.64%; P=0.434) that were comparable to those of the upfront surgery group. The neoadjuvant group experienced a shorter median postoperative hospital stay [10 days (IQR 7.8-13.0) versus 14 days (IQR 9.0-15.0); P=0.032]. The prevalence of satellite focus was lower in the neoadjuvant group (13.6% vs. 35%), although these findings did not achieve statistical significance, possibly a result of preoperative adjuvant therapy deactivating peritumoral microsatellite focus. Both groups exhibited similar tumor differentiation statuses and proportions of positive resection margins.

**Figure 2:**
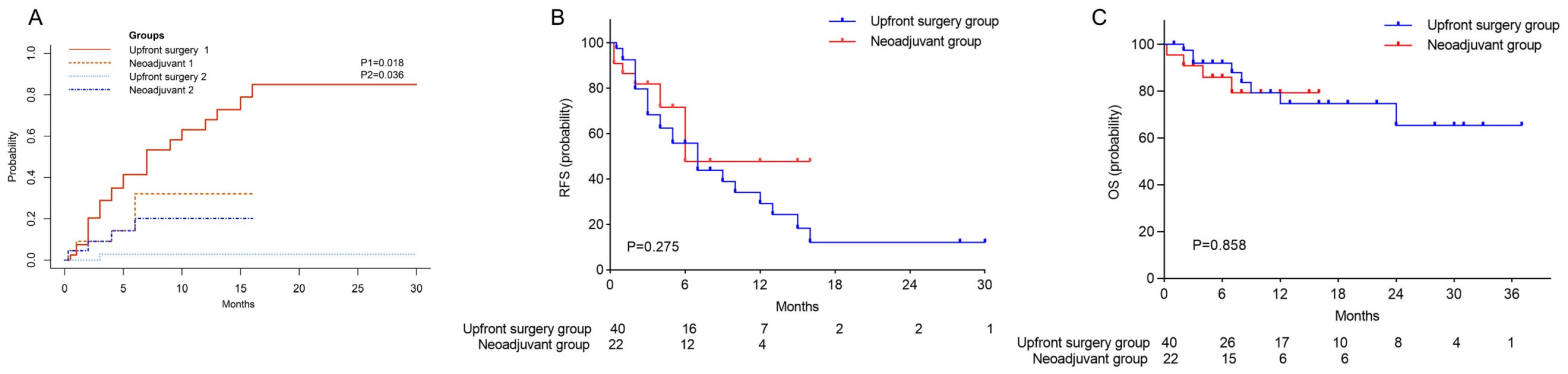
Survival analysis between the upfront surgery group and neoadjuvant group. (A) Cumulative incidence of postoperative tumor recurrence and nononcologic mortality between the two groups, 1 refers to tumor recurrence, and 2 refers to nononcologic mortality. (B) Kaplan-Meier Curve for RFS between the two groups. (C) Kaplan-Meier Curve for OS between the two groups. RFS, recurrence-free survival, OS, overall survival.

The median follow-up durations were 9 months (IQR 5.0-19.5) for the upfront surgery cohort and 8.5 months (IQR 5.8-13.8) for the neoadjuvant cohort. Given instances of nononcologic mortality among these patients, a multifactorial competing risks model was deployed to discern clinical factors influencing patient prognosis (Table 3). Preoperative adjuvant therapy followed by surgery markedly decreased the hazard of tumor recurrence (Hazard ratio [HR]=0.39, 95% CI 0.15-0.98; P=0.046), whereas larger tumor size (HR=1.13, 95% CI 1.01- 1.27; P=0.028) and R1 resection status (HR=4.61, 95%CI 1.55-13.75; P=0.006) were associated with a heightened hazard. Furthermore, neoadjuvant targeted immunotherapy preceding surgery resulted in substantially reduced postoperative tumor recurrence rates after both 3 months and 1 year (9% vs. 28.9% and 32.1% vs. 67.9%, respectively; P=0.018) (Figure 2A). However, the neoadjuvant cohort demonstrated a higher rate of 1-year nononcologic mortality (20.1% vs. 2.8%; P=0.036). There were no statistically significant differences in RFS and OS between the groups (Table 2; figures 2B, C). One patient from the neoadjuvant group experienced biliary leakage with subsequent biliary stricture, developed a secondary biliary infection, and ultimately succumbed to secondary pulmonary embolism and pulmonary heart disease. Additionally, a separate patient from the same group suffered from postoperative bile leakage and biliary stricture, endured recurrent biliary tract infections, and passed away from infectious shock. A further patient in the neoadjuvant group, notwithstanding an excellent PCR, faced multiple postoperative complications necessitating ongoing medical management, these included liver function impairment, massive thoracoabdominal effusion, gastrointestinal bleeding, liver abscess, gastrobiliary fistula, biliary stricture, and thrombocytopenia, that led to recurrent hospital admissions and substantially diminished quality of life.

## Discussion

The Chinese guidelines 4 and National Comprehensive Cancer Network (NCCN) guidelines concur that surgical resection, whether performed immediately (upfront surgery) or following preoperative adjuvant therapy, is the primary treatment for HCC with macrovascular invasion. The advent of immunotherapy has revolutionized the adjuvant treatment of HCC. Preoperative targeted immunotherapy allowed more patients with advanced disease to attempt radical surgical resection, thereby greatly prolonging the OS of advanced HCC 29, 30. Evidence from several cohorts illustrates that the combination of targeted therapy and immune therapy, optionally augmented by additional interventions, has yielded improved ORR of 30.0%-80.6% and increased conversion surgery rates of 10.0%-42.4% in patients with advanced HCC 24, 26, 31-33. However, little attention has been paid to the difference in postoperative outcomes between upfront surgery and surgical resection after neoadjuvant targeted immunotherapy. In this study, we compared the rate of perioperative complications, RFS and OS among HCC patients with macrovascular invasion who received upfront surgery or surgical resection subsequent to preoperative targeted immunotherapy.

HCC with macrovascular invasion is an aggressive tumor that presents formidable challenges to surgical resection due to its size and invasion on adjacent structures. Optimistically, the objectives of preoperative adjuvant therapy include tumor shrinkage and downgrading, thereby reducing the difficulty of subsequent surgery. However, therapy-induced liver function impairment and perihepatic adhesion, along with increased vessel fragility and subsequent intraoperative bleeding, pose additional challenges. As Zhou et al. reported, preoperative TACE alone did not improve surgical outcomes, but instead increased surgical difficulty and higher risk of postoperative liver failure 34. In our study, the upfront surgery group and neoadjuvant group had similar operative time, hepatic hilar occlusion time, and intraoperative blood loss, suggesting that the benefits and additional problems of preoperative adjuvant therapy seem to balance out, with no significant impact on the difficulty of subsequent surgery.

Preoperative adjuvant therapy resulted in increased incidence and volume of postoperative ascites, but perioperative blood transfusion rates, postoperative bilirubin, albumin, PT, bile leakages, and other major complications were comparable between the two groups, suggesting that although preoperative adjuvant therapy may induce some liver damage, the essential and synthetic functions of the liver remain generally preserved. Conversely, postoperative hospital stay was shortened for patients who received preoperative adjuvant therapy, indicating that the targeted immunotherapy-associated ascites had minor effects on postoperative recovery. Moreover, advancements in medical technology and enhanced postoperative care have greatly improved the recovery process, thereby reducing the length of postoperative hospital stays.

Our research suggests that preoperative targeted immunotherapy appears to potentially increase the risk of serious postoperative biliary complications, although there is no statistically significant difference in the incidence of biliary leakages between the two groups. Two patients from the neoadjuvant cohort experienced postoperative bile leakages that further led to life-endangering complications, while another developed a postoperative gastro-biliary fistula followed by a biliary stricture that significantly impaired the post-surgery quality of life. Mechanistically, preoperative adjuvant therapy, particularly immunotherapy, has been shown to damage hepatocytes and bile ducts, thereby compromising postoperative recovery 35-37. Severe biliary complications can critically impact patients' quality of life and potentially be life-threatening. In our study, bile leakages and subsequent complications partially contributed to the nononcologic mortality rate of neoadjuvant cohort, thus influencing the observed discrepancies in postoperative nononcologic mortality rates between the two cohorts. However, the robustness of this conclusion must be approached with caution, considering the limited sample size of our study and the presence of statistically nonsignificant trends.

Further Kaplan-Meier survival analysis revealed no significant difference in RFS and OS between the two groups. However, given the presence of nononcologic mortalities in both cohorts, we performed a competing risk survival analysis and found that preoperative adjuvant therapy significantly reduced the postoperative recurrence rate in HCC patients with macrovascular invasion, but also increased the risk of postoperative nononcologic mortality. The significant decrease in postoperative recurrence rate in neoadjuvant group patients could be attributable to the suppressive effects of the adjuvant therapy on satellite focus and oncologic microvascular thrombus. The increased incidence of nononcologic mortality in the neoadjuvant group may be related to liver function and biliary system damage by preoperative targeted immunotherapy. Two cases in the neoadjuvant group died from complications related to liver failure, while another two died due to further complications induced by postoperative bile leakages. In contrast, only one case in the upfront surgery group experiecnced nononcologic mortality due to postoperative liver failure. Barbier et al. performed preoperative adjuvant sorafenib therapy for advanced HCC and found that preoperative sorafenib treatment also increased the incidence of postoperative nononcologic mortality: four patients in the sorafenib group died due to postoperative liver failure, while only one patient in the control group died due to postoperative liver failure 38. Many literatures suggest that antiangiogenic drugs, ICIs, RT, and many other interventions can lead to elevated transaminases and bile duct injury 39, 40. Hepatoprotective drugs offered some protection, leading to normal values from biochemical tests. However, therapeutic damage to hepatic tissue and function may persist over extended periods, as illustrated by the ongoing postoperative complications observed in certain participants within this study.

Based on the aforementioned results and analysis, we believe that preoperative targeted immunotherapy is both safe and effective in clinical research. Compared to upfront surgery, patients receiving neoadjuvant targeted immunotherapy have a statistically significant increase in postoperative nononcologic mortality, but the difference is relatively small and may decrease with an increase in sample size. Numerous studies have shown that targeted immunotherapy is safe and well-tolerated 41, 42, and our two study groups did not have a significant difference in the occurrence of other major postoperative complications, indicating the safety of neoadjuvant targeted immunotherapy. Additionally, even though there is a slight increase in the risk of postoperative nononcologic mortality with neoadjuvant targeted immunotherapy, this risk is accidental and can be improved. With accumulated experience, we can reduce nononcologic mortality through improved targeted immunotherapy regimens, better preoperative evaluation, improved surgical skills, and enhanced postoperative supportive care regimens.

This study has potential limitations. Firstly, the inclusion criteria for the study might introduce bias, as the definition of vascular invasion includes both portal and hepatic veins, yet the subtype and extent of such invasions' influence on patient prognosis remain unexplored due to insufficient data. Secondly, the patient cohorts from four different medical centers in China, exhibited variation in indications for neoadjuvant targeted immunotherapy or upfront surgery, as well as inconsistencies in administered medications, contributing to increased heterogeneity. Thirdly, this is a retrospective study with small sample size, and prospective studies with larger sample size are needed to validate the effects of neoadjuvant targeted immunotherapy on short-term and long-term prognosis of HCC patients with macrovascular invasion. Lastly, considering the most of participant were of same gender (males) and of similar liver background disease (HBV). The results of this trial must have been affected following this deviation, especially that the number of all participants was relatively small.

## Conclusion

The finding of this study suggested that preoperative targeted immunotherapy may be a relatively safe and beneficial treatment for certain HCC patients with macrovascular invasion. While facilitating the potential for curative resection, this approach also reduces the incidence of postoperative tumor recurrence. Nonetheless, such preoperative intervention might lead to chronic hepatic and biliary injury, resulting in heightened nononcologic mortality. As a result, the adoption of preoperative targeted immunotherapy followed by surgical resection for HCC with macrovascular invasion necessitates careful consideration regarding patient selection, timing, and regimens. Given that this study is a retrospective study with a small sample size, a prospective study of a larger sample size would provide more confidence in these conclusions.

## Figures and Tables

**Table 1:** Baseline characteristics of the study cohorts

Parameters	Total (n=62)	Upfront surgery (n=40)	Neoadjuvant (n=22)	P-value
Gender, n (%)				0.908
Male	56 (90.3)	36 (90.0)	20 (90.9)	
Female	6 (9.7)	4 (10.0)	2 (9.1)	
Age, median (IQR), years	55.0 (45.8, 61.3)	56.0 (48.2, 62.8)	53.0 (43.0, 57.0)	0.102
HBV, n (%)	52 (83.9)	34 (85.0)	17 (77.27)	0.446
Antivirus, n (%)	17 (27.4)	6 (15.0)	11 (50.0)	0.003
Liver cirrhosis, n (%)	50 (80.6)	32 (80.0)	18 (81.8)	0.862
ECOG-PS, n (%)				0.082
0	39 (63.0)	22 (55.0)	17 (77.3)	
1	23 (37.0)	18 (45.0)	5 (22.7)	
Child-Pugh stage, n (%)				0.650
Grade A	58 (93.5)	37 (92.5)	21 (95.5)	
Grade B	4 (6.5)	3 (7.5)	1 (4.5)	
Basic bilirubin, median (IQR), umol/L	13.3 (10.9, 19.1)	15.1 (11.1, 21.9)	12.3 (10.4,15.23)	0.109
Basic albumin, median (IQR), g/L	39.0 (37.0, 41.1)	39.1 (37.1 42.0)	38.2 (36.0, 41.0)	0.247
Basic prothrombin time, median (IQR), s	12.1 (11.3, 13.0)	12.3 (11.4, 13.3)	11.9 (11.3, 12.4)	0.164
AFP level, n (%)				0.929
≤ 20 ng/L	23 (37.1)	15 (37.5)	8 (36.4)	
>20 ng/L	39 (62.9)	25 (62.5)	14 (63.6)	
Tumor diameter, median (IQR), cm	7.1 (5.0, 9.4)	6.8 (5.0, 9.5)	7.3 (5.0, 9.3)	0.906
Tumor number, n (%)				0.645
Single	39 (62.9)	26 (65.0)	13 (59.1)	
Multiple	23 (37.1)	14 (35.0)	9 (40.9)	
Tumor thrombus, n (%)				0.437
Portal vein	54 (87.1)	35 (87.5)	19 (86.4)	
Hepatic vein	6 (9.7)	3(7.5)	3 (13.6)	
Both	2 (3.2)	2 (5.0)	0 (0.0)	
Anatomic resection, n (%)	46 (74.2)	28 (70.0)	18 (81.8)	0.309
Hepatectomy, n (%)				0.176
Minor	38 (61.3)	27 (67.5)	11 (50.0)	
Major	24 (38.7)	13 (32.5)	11 (50.0)	

IQR, interquartile range; ECOG-PS, Eastern Cooperative Oncology Group Performance Status; HBV, hepatitis B virus; AFP, alpha-fetoprotein.

**Table 2:** Characteristics of surgical and postoperative features

Outcomes	Total (n=62)	Upfront surgery (n=40)	Neoadjuvant (n=22)	P-value
Operative time, median (IQR), min	267.5 (210.0, 300.0)	240.0 (202.5, 300.0)	292.0 (232.5, 343.8)	0.182
Hilar occlusion, median (IQR), min	22.5 (10.3, 34.0)	21.5 (12.3, 30.8)	31.5 (0.0, 62.3)	0.220
Intraoperative blood loss, median (IQR), ml	400.0 (200.0, 600.0)	400.0 (212.5.0, 600.0)	300.0 (175.0, 575.0)	0.270
Perioperative transfusion, n (%)	32 (51.6)	18 (45.0)	14(63.6)	0.160
Bilirubin, median (IQR), μmol/L	31.5 (23.9, 41.6)	31.4 (23.9, 43.2)	31.6 (25.9, 40.4)	1.000
Albumin, median (IQR), g/L	32.8 (29.5, 35.4)	31.8 (29.2, 34.8)	34.5 (30.2, 36.0)	0.233
Prothrombin time, median (IQR), s	14.0 (12.3, 15.7)	13.4 (11.9, 15.7)	14.7 (13.1, 15.5)	0.369
Clavien-Dindo classification, n (%)				0.699
grade 0/I	40 (64.5)	27 (67.5)	13 (59.1)	
grade II	14 (22.6)	9 (22.5)	5 (22.7)	
grade III	5(8.1)	3 (7.5)	2 (9.1)	
grade IV	2(3.2)	1 (2.5)	1 (4.5)	
grade V	1(1.6)	0 (0.0)	1 (4.5)	
Major complications, n (%)	8(12.9)	4 (10.0)	4 (18.2)	0.438
Bile leakage, n (%)	6 (9.7)	3 (7.5)	3 (13.64)	0.434
Ascites, n (%)	31 (50.0)	15 (37.5)	16 (72.73)	0.008
Postoperative hospital stay, median (IQR), days	12.0 (8.0, 15.0)	14.0 (9.0, 15.0)	10.0 (7.8, 13.0)	0.032
90-days-mortality, n (%)	5 (8.1)	3 (7.5)	2 (9.1)	0.826
Tumor differentiation, n (%)				0.637
Poor	25 (40.3)	17 (42.5)	8 (36.4)	
Moderately/Well	37 (59.7)	23 (57.5)	14 (63.6)	
Satellite foci, n (%)	17 (27.4)	14 (35.0)	3 (13.6)	0.071
Cutting edge, n (%)				0.650
R0	58 (93.5)	37 (92.5)	21 (95.5)	
R1	4 (6.5)	3 (7.5)	1 (4.5)	
Survival analysis				
RFS, mean (95%CI), month	11.5 (8.2, 14.9)	9.7 (6.3, 13.2)	9.6 (6.8, 12.5)	0.275
OS, mean (95%CI), month	28.2 (23.8, 32.6)	28.2 (23.0, 33.4)	13.5 (11.2, 15.7)	0.858

IQR, interquartile range; RFS, recurrence free survival; OS, overall survival.

**Table 4:** Neoadjuvant treatments based on targeted immunotherapy

Preoperative regimens	Cases	Duration, mean, months	Interval, mean, Days	PR	SD	PCR
Antiangiogenic+ICI	2	6.3	11	2/2	0/2	0/2
Antiangiogenic+ICI+HAIC	4	3.8	28	4/4	0/4	2/4
Antiangiogenic+ICI+TACE	8	3.7	18	7/8	1/8	1/8
Antiangiogenic+ICI+TACE+HAIC	1	4.8	12	1/1	0/1	0/1
Antiangiogenic+ICI+RT	3	3.5	12	3/3	0/3	0/3
Antiangiogenic+ICI+TACE+RT	4	4.2	19	4/4	0/4	0/4
Total	22	4.0 (3.0, 4.9)	18 (9.5, 27.0)	21/22	1/22	3/22

Duration, the time from the beginning to the end of preoperative adjuvant treatment; Interval, the time from the end of preoperative treatment to the date of surgery; PR, partial remission; SD, stable disease; PCR, pathologic complete response; ICI, immune checkpoint inhibitors; HAIC, hepatic arterial infusion chemotherapy; TACE, transarterial chemoembolization; RT, radiotherapy.

**Table 3:** A competing risk model of RFS

Parameters	HR	95% CI	P-value
Treatment groups (Neoadjuvant versus upfront surgery)	0.39	0.15 to 0.98	0.046
Antivirus treatment (yes versus no)	0.83	0.26 to 2.65	0.750
AFP (>20 ng/L versus ≤ 20 ng/L)	0.76	0.36 to 1.61	0.470
Tumor diameter	1.13	1.01 to 1.27	0.028
Tumor number (multiple versus single)	1.58	0.67 to 3.77	0.300
Anatomic resection (yes versus no)	0.45	0.17 to 1.18	0.100
Tumor differentiation (Moderately/Well versus poor)	1.18	0.44 to 3.13	0.740
Satellite foci (yes versus no)	0.98	0.45 to 2.13	0.960
Resection margin (R1 versus R0)	4.61	1.55 to 13.75	0.006

AFP, alpha-fetoprotein.
